# A calibrated Bayesian method for the stratified proportional hazards model with missing covariates

**DOI:** 10.1007/s10985-021-09542-4

**Published:** 2022-01-16

**Authors:** Soyoung Kim, Jae-Kwang Kim, Kwang Woo Ahn

**Affiliations:** Division of Biostatistics, Medical College of Wisconsin, Milwaukee, WI 53226-0509; Department of Statistics, Iowa State University, 2438 Osborn Dr Ames, IA 50011-1090; Division of Biostatistics, Medical College of Wisconsin, Milwaukee, WI 53226-0509

**Keywords:** Bayesian computation, Cox model, Missing data, Posterior distribution, Survival data

## Abstract

Missing covariates are commonly encountered when evaluating covariate effects on survival outcomes. Excluding missing data from the analysis may lead to biased parameter estimation and a misleading conclusion. The inverse probability weighting method is widely used to handle missing covariates. However, obtaining asymptotic variance in frequentist inference is complicated because it involves estimating parameters for propensity scores. In this paper, we propose a new approach based on an approximate Bayesian method without using Taylor expansion to handle missing covariates for survival data. We consider a stratified proportional hazards model so that it can be used for the non-proportional hazards structure. Two cases for missing pattern are studied: a single missing pattern and multiple missing patterns. The proposed estimators are shown to be consistent and asymptotically normal, which matches the frequentist asymptotic properties. Simulation studies show that our proposed estimators are asymptotically unbiased and the credible region obtained from posterior distribution is close to the frequentist confidence interval. The algorithm is straightforward and computationally efficient. We apply the proposed method to a stem cell transplantation data set.

## 1 Introduction

The proportional hazards (PH) model [9] is widely used to evaluate covariate effects on survival outcome. In many biomedical studies, covariate information is often incompletely observed for some subjects due to loss of follow-up, loss of hospital records, or study design. For example, Dreger el al. [11] studied 1,394 patients who were aged 18 years or older, had relapsed diffuse large B-cell lymphoma (DLBCL), and had undergone their first non-myeloablative or reduced-intensity conditioning allogeneic stem cell transplantation between 2008 and 2015. They compared the effect of the following donor types on overall survival: haploidentical family donors using post-transplant cyclophosphamide (PTCy), matched sibling donors (MSD) / matched unrelated donors (MUD) with or without T-cell depletion. There were missing records in hematopoietic cell transplant-comorbidity index. In addition, the PH assumption was not valid for remission status at time of transplant.

One simple way, but a commonly used method, to handle such incomplete data is excluding missing data from the analysis, which is called the complete-case (CC) method. However, the CC method may lead to biased parameter estimation when the missing mechanism is related to outcome variables. A more sensible way to handle missing data is using the propensity score weighting, which is based on a model for the response probability. Once the response probabilities are estimated, the inverse of the estimated response probability is applied as the weight for estimating parameters of interest.

Extensive work for the PH model with missing covariates has been done under the frequentist framework. Lin and Ying [23] proposed a pseudo-likelihood score function to handle missing covariates under the missing-completely-at-random assumption. Zhou and Pepe [44] and Chen and Little [5] proposed an estimated partial-likelihood method using auxiliary covariates and a nonparametric maximum likelihood method, respectively. Pugh et al. [29] proposed an inverse-probability-weighted (IPW) estimating equation when the missing mechanism is missing at random (MAR) in the sense of Rubin [31]. Wang and Chen [39] and Xu et al. [41] considered an augmented inverse probability weighting (AIPW) scheme [30] under the MAR assumption. Herring and Ibrahim [14] and Herring et al. [15] introduced Monte-Carlo-Expectation-Maximization-algorithm-based methods to handle missing covariates under the MAR assumption and non-ignorable missing, respectively. Multiple imputation for the PH model has also been explored [24,40,3]. However, all of these methods are limited to the non-stratified PH model. Because it is common that the PH assumption is not satisfied for some covariates [11,38,27] or stratified sampling is used for data collection [18,19], it is crucial to study the stratified PH model for missing data. In addition, many of these existing methods rely on Taylor expansion to study the asymptotic properties of the estimators and thus obtaining variance estimates of the estimators can be complicated in practice.

Besides the frequentist approaches, several Bayesian approaches were also proposed to handle missing covariates for survival data. Chen et al. [6] proposed a general class of informative priors for semi-parametric survival models incorporating a cure fraction. Yoo and Lee [42] extended the Bayesian adaptive B-spline estimation method of Sharef et al. [34] to the clustered survival data with missing covariates. Hemming and Hutton [13] considered Markov Chain Monte Carlo (MCMC) to handle missing covariates for the accelerated failure time model under the MAR assumption. For the PH model, Ibrahim et al. [17] and Bradshaw et al. [4] studied variable selection and non-ignorably missing time-varying covariates, respectively, under the MAR assumption. Chen et al. [8] considered covariates with detection limit. In general, the current Bayesian literature for missing covariates for the PH model requires complicated MCMC algorithms that generate correlated samples and the burn-in period. Finding straightforward and less computationally intensive methods for survival outcomes with missing data is desirable in practice. Furthermore, like the frequentist approaches discussed previously, these Bayesian methods did not consider the stratified PH model.

Sang and Kim [32] recently proposed an approximate Bayesian method to handle unit nonresponse for outcomes. Their Bayesian method is calibrated to the frequentist inference in that the credible region obtained from the posterior distribution asymptotically matches the frequentist confidence interval. Although their method is an approximate method based on the asymptotic normality of the score function, it is attractive in practice due to its simplicity and good performance in parameter estimation. More specifically, their algorithm generates independent samples for the posterior distribution of parameters and does not require MCMC for posterior computation.

Survival data often have multiple missing covariates. For example, Pidala et al. [26] studied the impact of DPB1 T-cell epitope matching on the hematopoietic cell transplantation outcomes for patients with leukemia and myelodysplastic syndrome. There were two important variables with missing values: DPB1 T-cell epitope matching and Karnofsky performance score (KPS). Existing literature considers one missing mechanism to handle these two missing covariates. However, the missing mechanism for DPB1 T-cell epitope matching could be different from one for KPS. In addition, cases having missing values in both variables could have a different missing mechanism from those with missing values in one of the two variables. This important aspect has been largely ignored in the current literature.

Motivated by Sang and Kim [32], we propose an approximate Bayesian approach for survival data with missing covariates. We consider the stratified PH model so that it can be used even for data with the non-proportional hazard structure. The proposed method uses two score equations, one from the stratified PH model and the other from the response model, to construct the likelihood part of the posterior distribution. With a flat prior, the credible interval from the posterior distribution asymptotically matches the confidence interval of the frequentist IPW approach. In contrast with the frequentist approach, the proposed method does not involve Taylor expansion. Sampling from the posterior distribution is straightforward to implement. We also propose new methods to take account of multiple covariates missing patterns. We study the asymptotic properties of the IPW estimators in frequentist inference for the stratified PH model with known and unknown missing mechanisms. Simulation studies and applications to the data of Dreger et al. [11] and Pidala et al. [26] are also provided.

## 2 Model and estimation

### 2.1 Model definitions and assumptions

Assume the full cohort consists of *n* subjects and there are *L* strata, where *L* is fixed. Let $T_{li}$ be the failure time, $C_{li}$ be the potential censoring time, and $Z_{li}={\left(Z_{li1},\ldots ,Z_{lip}\right)}^{T}$ be a p×1 time-independent covariate vector for subject *i* in stratum l for l=1,…,L, $i=1,\ldots ,n_{l}$, where *n_l_* is the number of subjects in stratum l. Let $X_{li}=\min\left(T_{li},C_{li}\right)$ denote the observed time in the full cohort and $\Delta_{li}=I\left(T_{li}\leq C_{li}\right)$ be an event indicator. The study period is [0,τ]. We consider the stratified PH model: for subject *i* in stratum *l,* the hazard function $\lambda_{li}\left(\cdot \right)$ associated with ***Z***_*li*_ is

(1) $$\lambda_{li}\left(t|Z_{li}\right)=\lambda_{0l}(t)e^{\beta_{0}^{T}Z_{li}},$$

where $\lambda_{0l}\left(t\right)$ is a baseline hazard function for stratum l and $\beta_{0}$ is an unknown parameter vector. We assume $T_{li}$ is independent of $C_{li}$ given $Z_{li}$ [9,10].

Next, we introduce notation and assumptions for missing covariates. Let $Z_{li}=\left(Z_{li}^{c},Z_{li}^{m}\right)$, where $Z_{li}^{c}$ and $Z_{li}^{m}$ are the complete covariate vector and the missing covariate vector for subject *i* in stratum *l*, respectively. Let $\xi_{li}$ be the observation indicator for subject *i* in stratum $l:\;\xi_{li}=1$ if $Z_{li}$ is fully observed and $\xi_{li}=0$ if some elements of $Z_{li}$ are missing. Assume $\left(T_{li},C_{li},Z_{li},\xi_{li}\right)$ for $i=1,\ldots ,n_{l}$ within stratum l are independently and identically distributed. In addition, $\left(T_{li},C_{li},Z_{li},\xi_{li}\right)$ and $\left(T_{l^{\prime }i^{\prime }},C_{l^{\prime }i^{\prime }},Z_{l^{\prime }i^{\prime }},\xi_{l^{\prime }i^{\prime }}\right)$ are assumed to be independent when $l\neq l^{\prime }$. The observed data for stratum l is $\left(X_{li},\Delta_{li},Z_{li}^{c},\xi_{li}\right)$ for $i=1,\ldots ,n_{l}$. Define $W_{li}=\left(X_{li},\Delta_{li},Z_{li}^{c}\right)$ for $i=1,\ldots ,n_{l}$ and l=1,…,L. We assume that $Z_{li}^{m}$ is missing at random in that the probability of observing missing covariates is conditionally independent of $Z_{li}^{m}$ given $W_{li}$. Let $N_{li}\left(t\right)=I\left(X_{li}\leq t,\Delta_{li}=1\right)$ be the counting process for the observed failure time, and $Y_{li}\left(t\right)=I\left(X_{li}\geq t\right)$ denote the at-risk indicator for subject *i* in stratum *l,* where I(⋅) is an indicator function.

### 2.2 Inverse Probability Weighted Estimators

The inverse probability weighted (IPW) estimator based on Horvitz and Thompson [16] adjusts for missing covariates by using the inverse of the response probability as the weight [39,41]. We assume the $\xi_{li}$ within stratum *l* is independently generated from a Bernoulli distribution and allow a different intercept for each stratum:

(2) $$\pi_{li}=\mathrm{Pr}\left(\xi_{li}=1|W_{li}\right)=\frac{\exp\left(\phi^{T}\omega_{li}\right)}{1+\exp\left(\phi^{T}\omega_{li}\right)},$$

where $\omega_{li}={\left(1,I\left(l=2\right),\ldots ,I\left(l=L\right),W_{li}^{T}\right)}^{T}$. Let $\phi_{0}$ be the true parameter vector of ϕ. To obtain the IPW estimator, we consider

(3) $$U_{1,n}(\beta ,\phi )=\frac{1}{n}\overset{L}{\underset{l=1}{\sum }}\overset{n_{l}}{\underset{i=1}{\sum }}\frac{\xi_{li}}{\pi_{li}}{\int^{\text{​}}}_{0}^{\tau }\left\{Z_{li}-\frac{S_{l}^{\left(1\right)}(\beta ,t)}{S_{l}^{\left(0\right)}(\beta ,t)}\right\}\;dN_{li}(t)=0,$$

(4) $$U_{2,n}(\phi )=\frac{1}{n}\overset{L}{\underset{l=1}{\sum }}\overset{n_{l}}{\underset{i=1}{\sum }}\left\{\xi_{li}-\pi_{li}\right\}\omega_{li}^{T}=0,$$

where $S_{l}^{\left(d\right)}\left(\beta ,t\right)=n^{-1}\sum_{i=1}^{n_{l}}\xi_{li}\pi_{li}^{-1}Y_{li}\left(t\right)Z_{li}^{⊗d}e^{\beta^{T}Z_{li}}$ for d=0,1 and $a^{⊗0}=1$, $a^{⊗1}=a$, $a^{⊗2}=aa^{T}$. The two functions $U_{1,n}\left(\beta ,\phi \right)$ and $U_{2,n}\left(\phi \right)$ are the score functions from the stratified PH model and the logistic regression, respectively. For the frequentist IPW approach under the MAR assumption, one first estimates response probabilities $\pi_{li}’s$ by solving (4) and then plugs the estimated $\pi_{li}’s$ into (3) to estimate ***β***. Let the solution to (3) be $\hat{\beta }$. In practice, one relies on Taylor expansion to obtain the asymptotic normality of $\hat{\beta }$ [41].

### 2.3 Approximate Bayesian approach

We propose an approximate Bayesian approach in this section. Define $\theta ={\left(\beta^{T},\phi^{T}\right)}^{T}$ and $U_{n}\left(\theta \right)={\left(U_{1,n}^{T}\left(\beta ,\phi \right),U_{2,n}^{T}\left(\phi \right)\right)}^{T}$. Let $\hat{\theta }$ be the solution to $U_{n}\left(\theta \right)=0$. Instead of directly generating the posterior distribution p(θ|data), similarly to Soubeyrand and Haon-Lasportes [35], we use an approximation to p(θ|data), that is, $p\left(\theta |\hat{\theta }\right)$ as follows:

$$p(\theta |\hat{\theta })\propto p(\hat{\theta }|\theta )p(\theta ),$$

where $p\left(\hat{\theta }|\theta \right)$ is the sampling distribution of $\hat{\theta }$ and p(θ) is the prior distribution for θ. However, studying $p\left(\hat{\theta }|\theta \right)$ requires Taylor expansion, as in Theorem 1.

To avoid Taylor expansion, instead of generating from $p\left(\theta |\hat{\theta }\right)$, we alternatively consider

(5) $$p\left(\theta |U_{n}\right)\propto p\left(U_{n}(\theta )|\theta \right)p(\theta ),$$

where $p\left(U_{n}\left(\theta \right)|\theta \right)$ is the sampling distribution of $U_{n}\left(\theta \right)$. To generate samples from (5), we consider a one-to-one transformation T:θ→η such that $\eta =E\left(U_{n}|\theta \right)$. Then, we generate $\eta^{*}$ from $p\left(\eta |U_{n}\right)$ and obtain $\theta^{*}=T^{-1}\left(\eta^{*}\right)$ as samples for the posterior distribution in (5). As in the next section, under some regularity conditions, the asymptotic distribution of $U_{n}$ is

(6) $$\sqrt{n}\left\{U_{n}(\theta )-\eta (\theta )\right\}|\theta \overset{d}{\to }N(0,\Sigma ),$$

where $\overset{d}{\to }$ is convergence in distribution and Σ is the asymptotic covariance matrix of the joint score functions. Since the transformation T:θ→η is one-to-one, (6) is equivalent to

(7) $$\sqrt{n}\left\{U_{n}\left(T^{-1}\eta \right)-\eta \right\}|\eta \overset{d}{\to }N(0,\Sigma ).$$

Then, the posterior distribution of ***η*** given ***U***_*n*_ is

(8) $$p\left(\eta |U_{n}\right)\propto p\left(U_{n}|\eta \right)p(\eta ),$$

where $p\left(U_{n}|\eta \right)$ is the density of the limiting distribution in (7). Equation (8) shows an important relationship between the frequentist IPW approach and the Bayesian approach. Under a flat prior for p(η) and the sufficient conditions for (7), we can approximate the posterior distribution $p\left(\eta |U_{n}\right)$ as follows:

(9) $$p\left(\eta |U_{n}\right)∼N\left(U_{n},\Sigma /n\right).$$

As shown in the Appendix, the estimator of ***Σ***, $\hat{\Sigma }$, can be obtained by

$$\frac{\hat{\Sigma }}{n}=\left(\begin{array}{cc} \hat{Var}\left(U_{1}\right) & \hat{Cov}\left(U_{1},U_{2}\right)\\ \hat{Cov}\left(U_{1},U_{2}\right) & \hat{Var}\left(U_{2}\right) \end{array}\right),\;\hat{Var}\left\{U_{1}(\beta ,\phi )\right\}=\frac{1}{n^{2}}\overset{L}{\underset{l=1}{\sum }}\overset{n_{l}}{\underset{i=1}{\sum }}\frac{\xi_{li}}{\pi_{li}^{2}}\int_{0}^{\tau }{\left[\left\{Z_{li}-\frac{S_{l}^{\left(1\right)}(\beta ,t)}{S_{l}^{\left(0\right)}(\beta ,t)}\right\}\hat{M_{li}}(t)\right]}^{⊗2},\;\hat{Var}\left(U_{2}\right)=\frac{1}{n^{2}}\overset{L}{\underset{l=1}{\sum }}\overset{n_{l}}{\underset{i=1}{\sum }}\pi_{li}\left(1-\pi_{li}\right)\omega_{li}\omega_{li}^{T},\;d{\hat{\Lambda }}_{0l}(t)=\overset{n_{l}}{\underset{i=1}{\sum }}dN_{li}(t)/\left\{n_{l}S_{l}^{\left(0\right)}(\beta ,t)\right\},\;\hat{Cov}\left(U_{1},U_{2}\right)=\frac{1}{n^{2}}\overset{L}{\underset{l=1}{\sum }}\overset{n_{l}}{\underset{i=1}{\sum }}\frac{\xi_{li}\left(1-\pi_{li}\right)}{\pi_{li}}\int_{0}^{\tau }\left\{Z_{li}-\frac{S_{l}^{\left(1\right)}(\beta ,t)}{S_{l}^{\left(0\right)}(\beta ,t)}\right\}\;dM_{li}(t)\times \omega_{li}^{T},\;{\hat{\Sigma }}_{c}=\hat{Var}\left(U_{1}\right)-\hat{Cov}\left(U_{1},U_{2}\right){\hat{Var}}^{-1}\left(U_{2}\right)\hat{Cov}{\left(U_{1},U_{2}\right)}^{T},\;d{\hat{M}}_{li}(t)=dN_{li}(t)-Y_{li}(t)e^{\beta^{T}Z_{\mathrm{li}}}\;d{\hat{\Lambda }}_{0l}(t).$$

Under the flat prior for p(η), we propose the following algorithm to generate samples from the posterior distribution $p\left(\theta |\hat{\theta }\right)$ as follows:

1. Generate $\eta_{2}^{*}$ from the approximate posterior distribution $p\left(\eta_{2}|U_{2,n}=0\right)$, that is, $N\left(0,\hat{Var}\left(U_{2}\right)\right)$.
2. Solve $U_{2,n}\left(\phi \right)=\eta_{2}^{*}$ with respect to ϕ to obtain $\phi^{*}$.
3. Generate $\eta_{1}^{*}$ from the approximate posterior distribution $p\left(\eta_{1}|U_{1,n}\left(\phi^{*}\right)=0\right)$, that is, $N\left(0,{\hat{\Sigma }}_{c}\right)$.
4. Solve $U_{1,n}\left(\beta \right)=\eta_{1}^{*}$ with respect to β to obtain $\beta^{*}$.
5. Repeat above steps.

The Newton-Raphson algorithm or a root-finding algorithm can be used to solve $U_{n}\left(\theta \right)=\eta^{*}$ in Step 2 and Step 4. Thus, the algorithm is straightforward to implement. Based on our simulation results, 1000 repetitions appear to be enough for statistical inference. Using the above algorithm, independent samples from the approximate posterior distribution $p\left(\theta |\hat{\theta }\right)$ are generated and thus there is no burn-in period.

### 2.4 Estimators with multiple missing covariates patterns

The methods in Sections 2.2 and 2.3 consider a single missing mechanism. There are not directly applicable when there are multiple missing covariates having different missing patterns. We propose estimators for survival data with multiple missing patterns in this section. We describe the proposed method for the stratified PH model with two missing covariates and two strata for simplicity. Suppose two covariates, $Z_{1i}^{m}$ and $Z_{2i}^{m}$, are subject to missingness for individual *i*. Let $\eta_{ki}=1$ if $Z_{ki}^{m}$ is observed and $\eta_{ki}=0$ if $Z_{ki}^{m}$ is missing for *k* = 1, 2. Denote $Z={\left(Z_{1}^{m},Z_{2}^{m}\right)}^{T}$ and $O={\left(\Delta ,X,{\left(Z^{c}\right)}^{T}\right)}^{T}$, where $Z^{c}$ is a completely observed covariate vector. We divide the data into 4 groups: i) both $Z_{1}^{m}$ and $Z_{2}^{m}$ are observed; ii) $Z_{1}^{m}$ is observed and $Z_{2}^{m}$ is missing; iii) $Z_{1}^{m}$ is missing and $Z_{2}^{m}$ is observed; and iv) both $Z_{1}^{m}$ and $Z_{2}^{m}$ are missing. Let $\omega_{10}={\left(1,Z_{1}^{m},O^{T}\right)}^{T}$, $\omega_{01}={\left(1,Z_{2}^{m},O^{T}\right)}^{T}$, and $\omega_{00}={\left(1,O^{T}\right)}^{T}$. Thus, $\omega_{10}$, $\omega_{01}$, and $\omega_{00}$ correspond to ii), iii), and iv), respectively. Define

(10) $$\begin{array}{c} r_{11}(Z,O)=1,\;\;\;\;\;\;\text{if}\;\eta_{1}=1,\eta_{2}=1,\\ r_{10}(Z,O)=\exp\left(\phi_{10}^{T}\omega_{10}\right),\;\text{if}\;\eta_{1}=1,\eta_{2}=0,\\ r_{01}(Z,O)=\exp\left(\phi_{01}^{T}\omega_{01}\right),\;\text{if}\;\eta_{1}=0,\eta_{2}=1,\\ r_{00}(Z,O)=\exp\left(\phi_{00}^{T}\omega_{00}\right),\;\text{if}\;\eta_{1}=0,\eta_{2}=0, \end{array}$$

where

$$\phi_{10}^{T}\omega_{10}=\phi_{10,0}+\phi_{10,1}Z_{1}^{m}+\phi_{10,2}\Delta +\phi_{10,3}X+\phi_{10,4}^{T}Z^{c}+\phi_{10,5}^{T}I(\mathrm{stratum}=2),\;\phi_{01}^{T}\omega_{01}=\phi_{01,0}+\phi_{01,1}Z_{2}^{m}+\phi_{01,2}\Delta +\phi_{01,3}X+\phi_{01,4}^{T}Z^{c}+\phi_{01,5}^{T}I(\mathrm{stratum}=2),\;\phi_{00}^{T}\omega_{00}=\phi_{00,0}+\phi_{00,1}\Delta +\phi_{00,2}X+\phi_{00,3}^{T}Z^{c}+\phi_{00,4}^{T}I(\mathrm{stratum}=2).$$

Equation (10) satisfies the MAR assumption. Model (10) is a model for the ratio of the missing probability to the baseline:

$$r_{ab}(Z,O)=\frac{P\left(\eta_{1}=a,\eta_{2}=b|Z,O\right)}{P\left(\eta_{1}=1,\eta_{2}=1|Z,O\right)}.$$

A similar idea has been considered in Sun and Tchetgen Tchetgen [36]. Then, the propensity score is

$$\pi_{ab}=P\left(\eta_{1}=a,\eta_{2}=b|Z,O\right)=\frac{r_{ab}(Z,O)}{\sum_{a=0}^{1}\sum_{b=0}^{1}r_{ab}(Z,O)}.$$

Let $\phi_{0}$ be the true parameter vector of $\phi ={\left(\phi_{10}^{T},\phi_{01}^{T},\phi_{00}^{T}\right)}^{T}$. To obtain the IPW estimator, we consider

(11) $$U_{1,n}^{m}\left(\beta_{m},\phi \right)=\frac{1}{n}\overset{L}{\underset{l=1}{\sum }}\overset{n_{l}}{\underset{i=1}{\sum }}\frac{\xi_{li}}{\pi_{11,li}}\int_{0}^{\tau }\;\left\{Z_{li}-\frac{S_{l,m}^{\left(1\right)}\left(\beta_{m},t\right)}{S_{l,m}^{\left(0\right)}\left(\beta_{m},t\right)}\right\}\;dN_{li}(t)=0,$$

(12) $$U_{2,n}^{m}(\phi )={\left\{U_{10,n}^{T}\left(\phi_{10}\right),U_{01,n}^{T}\left(\phi_{01}\right),U_{00,n}^{T}\left(\phi_{00}\right)\right\}}^{T}=0,$$

$$U_{ab,n}\left(\phi_{ab}\right)=\frac{1}{N_{ab}}\overset{L}{\underset{l=1}{\sum }}\overset{n_{l}}{\underset{i=1}{\sum }}\eta_{ab,li}\left\{\xi_{li}-\frac{\pi_{11,li}}{\pi_{11,li}+\pi_{ab,li}}\right\}\omega_{ab,li}^{T},$$

where $\xi_{li}=I\left(\eta_{1}=1,\eta_{2}=1\right)$, $\eta_{ab,li}=I\left(\eta_{1li}=a,\eta_{2li}=b\;\text{or}\;\eta_{1li}=1,\eta_{2li}=1\right)$, $S_{l,m}^{\left(k\right)}\left(\beta_{m},t\right)=n_{l}^{-1}\sum_{i=1}^{n_{l}}\xi_{li}\pi_{11,li}^{-1}Y_{li}\left(t\right)Z_{li}^{⊗k}e^{\beta_{l,m}^{T}Z_{li}}$, and $N_{ab}$ is the subgroup sample size with $\eta_{1}=a$ and $\eta_{2}=b$. Let the solution to (11) be ${\hat{\beta }}_{m}$. Similarly to Section 2.3, the estimator of $\Sigma^{m}$, ${\hat{\Sigma }}^{m}$, can be obtained by

$$\frac{{\hat{\Sigma }}^{m}}{n}=\left(\begin{array}{cc} \hat{Var}\left(U_{1}^{m}\right) & \hat{Cov}\left(U_{1}^{m},U_{2}^{m}\right)\\ \hat{Cov}\left(U_{1}^{m},U_{2}^{m}\right) & \hat{Var}\left(U_{2}^{m}\right) \end{array}\right),\;\hat{Var}\left\{U_{1}^{m}(\beta ,\phi )\right\}=\frac{1}{n^{2}}\overset{L}{\underset{l=1}{\sum }}\overset{n_{l}}{\underset{i=1}{\sum }}\frac{\xi_{li}}{\pi_{11,li}^{2}}\int_{0}^{\tau }\;{\left[\left\{Z_{li}-\frac{S_{l,m}^{\left(1\right)}(\beta ,t)}{S_{l,m}^{\left(0\right)}(\beta ,t)}\right\}\;d{\hat{M}}_{li}(t)\right]}^{⊗2},\;\hat{Var}\left(U_{ab}\right)=\frac{1}{N_{ab}^{2}}\overset{L}{\underset{l=1}{\sum }}\overset{n_{l}}{\underset{i=1}{\sum }}\eta_{ab,li}\;{\left(\xi_{li}-\frac{\pi_{11,li}}{\pi_{11,li}+\pi_{ab,li}}\right)}^{2}\omega_{ab,li}\omega_{ab,li}^{T},\;d{\hat{\Lambda }}_{l0}(t)=\overset{n_{l}}{\underset{i=1}{\sum }}dN_{li}(t)/\left\{nS_{l,m}^{\left(0\right)}(\beta ,t)\right\},d{\hat{M}}_{li}(t)=dN_{li}(t)-Y_{li}(t)e^{\beta^{T}Z_{\mathrm{li}}}d{\hat{\Lambda }}_{l0}(t)\;\hat{Cov}\left(U_{1}^{m},U_{2}^{m}\right)={\left\{U_{1,n}^{m}\right\}}^{T}U_{2,n}^{m}.$$

Step 1 - Step 5 in Section 2.3 can be similarly applicable to the approximate Bayesian approach for multiple missing patterns.

## 3 Asymptotic properties

We now study the asymptotic properties of $p\left(\theta |\hat{\theta }\right)$ in this section. To establish the consistency and the asymptotic normality of the IPW estimator for the stratified PH model, we assume the following conditions:

C1 $\text{P}\left\{Y_{li}\left(t\right)=1\right\}>0$ for t∈[0,τ], l=1,…,L and $i=1,\ldots ,n_{l}$;

C2 $\left|Z_{lik}\left(0\right)\right|+\int_{0}^{\tau }\left|dZ_{lik}\left(t\right)\right|<D_{z}<\infty$, l=1,…,L, $i=1,\ldots ,n_{l}$ and k=1,…,p almost surely where $D_{z}$ is a constant;

C3 For *d* = 0, 1, 2, there exists a neighborhood B of $\beta_{0}$ such that $s_{l}^{\left(d\right)}\left(\beta ,t\right)$ is continuous and $\sup_{t\in \left[0,\tau \right],\beta \in B}\left\|S_{l}^{\left(d\right)}\left(\beta ,t\right)-s_{l}^{\left(d\right)}\left(\beta ,t\right)\right\|\overset{p}{\to }0$ for l=1,…,L, where $\overset{p}{\to }$ denotes convergence in probability;

C4 The matrix $I_{l}\left(\beta \right)=\int_{0}^{\tau }v_{l}\left(\beta ,t\right)s_{l}^{\left(0\right)}\left(\beta ,t\right)\lambda_{0l}\left(t\right)dt$ is positive definite for l=1,…,L, where $v_{l}\left(\beta ,t\right)=s_{l}^{\left(2\right)}\left(\beta ,t\right)/s_{l}^{\left(0\right)}\left(\beta ,t\right)-e_{l}{\left(\beta ,t\right)}^{⊗2}$ and $e_{l}\left(\beta ,t\right)=s_{l}^{\left(1\right)}\left(\beta ,t\right)/s_{l}^{\left(0\right)}\left(\beta ,t\right)$;

C5 The matrix $V_{l}^{\phi }$ is positive definite and $\pi_{li}\geq \epsilon >0$ for $i=1,\ldots ,n_{l}$ and l=1,…,L, where $V_{l}^{\phi }=E{\left\{\left(\xi_{l1}-\pi_{l1}\right)\omega_{l1}^{T}\right\}}^{⊗2}$;

C6 For all β∈B, t∈[0,τ], $S_{l}^{\left(1\right)}\left(\beta ,t\right)=\partial S_{l}^{\left(0\right)}\left(\beta ,t\right)/\partial \beta$, and $S_{l}^{\left(2\right)}\left(\beta ,t\right)=\partial^{2}S_{l}^{\left(0\right)}\left(\beta ,t\right)/\partial \beta \partial \beta^{T}$, where $S_{l}^{\left(d\right)}\left(\beta ,t\right)$, d=0,1,2 are continuous functions of β∈B uniformly in t∈[0,τ] and are bounded on B×[0,τ], $s_{l}^{\left(0\right)}$ is bounded away from zero on B×[0,τ];

C7 $\int_{0}^{\tau }\lambda_{0l}\left(t\right)dt<\infty$ for l=1,…,L;

C8 $\lim_{n\to \infty }n_{l}/n=q_{l}$, where $q_{l}\in \left(0,1\right)$ for all l=1,…,L;

C9 As n→∞, $\sup_{\theta \in \Theta }\left\|U_{n}\left(\theta \right)-\eta \left(\theta \right)\right\|\overset{p}{\to }0$, where Θ is the parameter space;

C10 The map $\theta \mapsto U_{n}\left(\theta \right)$ is continuous and has exactly one zero $\hat{\theta }$ with probability one;

C11 Equation η(θ)=0 has exactly one root at $\theta =\theta_{0}$;

C12 There exists a neighborhood of $\theta_{0}$, denoted by $J_{n}\left(\theta_{0}\right)$, on which with probability one all $U_{n}\left(\theta \right)$ are continuously differentiable and the Jacobian $\partial U_{n}\left(\theta \right)/\partial \theta$ converges uniformly to a non-stochastic limit which is non-singular. Here, $J_{n}\left(\theta_{0}\right)$ is a ball with center $\theta_{0}$ and radius $r_{n}$ satisfies $r_{n}\to \infty$ and $r_{n}\sqrt{n}\to \infty$;

C13 For any $\theta \in J_{n}\left(\theta_{0}\right)$, given θ:

$$\sqrt{n}\left\{U_{n}(\theta )-\eta (\theta )\right\}\overset{d}{\to }N(0,\Sigma (\theta ))$$

holds for some $\Sigma \left(\theta \right)=Var\left\{\sqrt{n}U_{n}\left(\theta \right)|\theta \right\}$ that is positive definite and independent of *n.*

Conditions C1–C8 are the standard conditions for the consistency and asymptotic normality of $\hat{\beta }$ [1,41]. Conditions C9–C13 are needed for the asymptotic properties of the joint IPW estimator. More specifically, as long as the samples satisfy some moment conditions, condition C9 holds. Conditions C10 and C11 ensure the existence and uniqueness of the solutions to $U_{n}=0$. Condition C12 regulates the derivatives of $U_{n}$ and ensures its covariance converges. Condition C13 provides the asymptotic distribution for the estimating equation. The proof of C13 can be found in Theorem 6 of Yuan and Jennrich (1998) [43], where Yuan and Jennrich (1998) [43] studied the large sample properties including the existence, strong consistency, and asymptotic normality of the estimators generated from samples that are not necessarily identically distributed under very general assumptions. Under Conditions C1-C13 we can show $\hat{\theta }$ is a consistent estimator for θ and asymptotically normally distributed with mean **0** and covariance matrix $B^{-1}\left(\theta_{0}\right)\Sigma \left(\theta_{0}\right)B^{-1}\left(\theta_{0}\right)$ where B(θ)=∂η(θ)/∂θ.

Following Sang and Kim [32], we assume the following conditions to establish the posterior consistency and asymptotic normality.

C14 The prior η↦π(η) is positive and Lipschitz continuous over the parameter space;

C15 For $\theta \in J_{n}\left(\theta_{0}\right)$, the variance estimator $\hat{\Sigma }\left(\theta \right)$ satisfies $\hat{\Sigma }\left(\theta \right)=\Sigma \left(\theta \right)\left\{1+o_{p}\left(1\right)\right\}$ where $\hat{\Sigma }\left(\theta \right)$ is provided in the Appendix;

C16 For any $\theta \in J_{n}\left(\theta_{0}\right)$, the mapping $\theta \mapsto {\left|\Sigma \left(\theta \right)\right|}^{-1}$ is Lipschitz continuous. Also, the mapping $\theta \mapsto x^{T}{\left\{\Sigma \left(\theta \right)\right\}}^{-1}x$ is Lipschitz continuous in the sense that there exists a constant C(x) satisfying $\left\|x^{T}{\left\{\Sigma \left(\theta_{1}\right)\right\}}^{-1}x-x^{T}{\left\{\Sigma \left(\theta_{2}\right)\right\}}^{-1}x\right\|\leq C\left(x\right)\left\|\theta_{1}-\theta_{2}\right\|$, for any $\theta_{1},\theta_{2}\in J_{n}\left(\theta_{0}\right)$, for all $x\in R^{p}$, where p=dim(Z). And C(x) is also Lipschitz continuous;

C17 $\theta \mapsto U_{n}\left(\theta \right)$ and θ↦η(θ) are one-to-one functions for any $\theta \in J_{n}\left(\theta_{0}\right)$. Also θ↦η(θ) is Lipschitz continuous.

Condition C14 is a standard assumption for the prior and the flat prior satisfies this condition. Condition C15 implies the covariance estimator should be consistent. Conditions C16 to C17 are the sufficient conditions for the posterior distribution to be approximated by the proposed method. Soubeyrand and Haon-Lasportes [35] also used similar conditions to C14 and C16 to justify their approximate Bayesian computation methods. All the conditions can be easily satisfied if we assume covariance estimator is Lipschitz continuous in ***θ*** and has bounded eigenvalues as discussed in Sang and Kim [32].

Similarly to Xu et al. [41], we can establish the following asymptotic property for $\hat{\beta }$ under the stratified PH model:

**Theorem 1** *Assume Conditions C1-C8 in* Section 3.

*1. Assume* $\pi_{li}$ *is unknown and correctly specified. Then*, $\hat{\beta }$ *is consistent for* β, and $\sqrt{n}\left(\hat{\beta }-\beta_{0}\right)$ *is asymptotically normally distributed with mean* **0** *and covariance matrix*

(13) $$V_{\beta }={\left\{I\left(\beta_{0}\right)\right\}}^{-1}\left\{\Sigma^{\beta_{0}}-\Sigma^{\phi_{0}\beta_{0}}\right\}{\left\{I\left(\beta_{0}\right)\right\}}^{-1},$$

*where*

$$I\left(\beta \right)=\sum_{l=1}^{L}q_{l}I_{l}\left(\beta \right),\;I_{l}\left(\beta \right)=\int_{0}^{\tau }v_{l}\left(\beta ,t\right)s_{l}^{\left(0\right)}\left(\beta ,t\right)\lambda_{0l}\left(t\right)dt,\;v_{l}\left(\beta ,t\right)=s_{l}^{\left(2\right)}\left(\beta ,t\right)/s_{l}^{\left(0\right)}\left(\beta ,t\right)-e_{l}{\left(\beta ,t\right)}^{⊗2},\;e_{l}\left(\beta ,t\right)=s_{l}^{\left(2\right)}\left(\beta ,t\right)/s_{l}^{\left(0\right)}\left(\beta ,t\right),\;s_{l}^{\left(d\right)}\left(\beta ,t\right)=E\left\{S_{l}^{\left(d\right)}\left(\beta ,t\right)\right\}\;for\;d=0,1,2,\;\Sigma^{\beta }=\sum_{l=1}^{L}q_{l}E{\left[\frac{\xi_{l1}}{\pi_{l1}}\int_{0}^{\tau }\left\{Z_{l1}-e_{l1}\left(\beta ,t\right)dM_{l1}\left(t\right)\right\}\right]}^{⊗2},\;\Sigma^{\phi \beta }=\sum_{l=1}^{L}q_{l}V_{l}^{\phi \beta }V_{l}^{\phi }{\left(V_{l}^{\phi \beta }\right)}^{T},\;V_{l}^{\phi \beta }=E\;\left[\frac{\xi_{l1}}{\pi_{l1}^{2}}\int_{0}^{\tau }\left\{Z_{l1}-e_{l1}\left(\beta ,t\right)dM_{l1}\left(t\right)\right\}\frac{\partial }{\partial \phi^{T}}\pi_{l1}\right],\;V_{l}^{\phi }=E{\left\{\left(\xi_{l1}-\pi_{l1}\right)\omega_{l1}^{T}\right\}}^{⊗2},\;dM_{li}\left(t\right)=dN_{li}\left(t\right)-Y_{li}\left(t\right)\exp\left(\beta^{T}Z_{li}\right)d\Lambda_{0l}\left(t\right),\;\underset{n\to \infty }{\lim}n_{l}/n=q_{l}.$$

*2. If π_li_ is known*, $\sqrt{n}\left(\hat{\beta }-\beta_{0}\right)$ *is asymptotically normally distributed with mean* **0** *and covariance matrix* ${\left\{I\left(\beta_{0}\right)\right\}}^{-1}\Sigma^{\beta_{0}}{\left\{I\left(\beta_{0}\right)\right\}}^{-1}$.

Its proof is a straightforward extension of Theorem 2 of Xu et al. [41] to the stratified PH model and thus omitted. Using (13), one can develop a plug-in variance estimator of $\hat{\beta }$, but it can involve a quite computation.

Next we have the following asymptotic property for estimators from the stratified PH model with two missing covariates having multiple missing patterns as in Section 2.4.

**Theorem 2** *Assume Conditions C1-C8 in* Section 3.

*1. Assume* $\pi_{ab,li}$ is unknown and correctly specified. Then, ${\hat{\beta }}_{m}$ is consistent for $\beta_{m}$, and $\sqrt{n}\left({\hat{\beta }}_{m}-\beta_{m0}\right)$ *is asymptotically normally distributed with mean* **0** *and covariance matrix*

$$V_{\beta }^{m}={\left\{I_{m}\left(\beta_{m0}\right)\right\}}^{-1}\left\{\Sigma_{m}^{\beta_{m0}}-\Sigma_{m}^{\phi_{m0}\beta_{m0}}\right\}{\left\{I_{m}\left(\beta_{m0}\right)\right\}}^{-1},$$

*where*

$$I_{m}\left(\beta \right)=\sum_{l=1}^{L}q_{l}\int_{0}^{\tau }v_{lm}\left(\beta ,t\right)s_{l,m}^{\left(0\right)}\left(\beta ,t\right)\lambda_{l0}\left(t\right)dt,\;v_{lm}\left(\beta ,t\right)=s_{l,m}^{\left(2\right)}\left(\beta ,t\right)/s_{l,m}^{\left(0\right)}\left(\beta ,t\right)-e_{l,m}{\left(\beta ,t\right)}^{⊗2},\;e_{l,m}\left(\beta ,t\right)=s_{l,m}^{\left(1\right)}\left(\beta ,t\right)/s_{l,m}^{\left(0\right)}\left(\beta ,t\right),\;s_{l,m}^{\left(d\right)}\left(\beta ,t\right)=E\left\{S_{l,m}^{\left(d\right)}\left(\beta ,t\right)\right\}\;for\;d=0,1,2,\;\Sigma_{m}^{\beta }=\sum_{l=1}^{L}q_{l}E\;{\left[\frac{\xi_{l1}}{\pi_{11,l1}}\int_{0}^{\tau }\left\{Z_{l1}-e_{l,m}\left(\beta ,t\right)dM_{l1}\left(t\right)\right\}\right]}^{⊗2},\;\Sigma_{m}^{\phi \beta }=V_{m}^{\phi \beta }V_{m}^{\phi }{\left(V_{m}^{\phi \beta }\right)}^{T},\;V_{m}^{\phi \beta }=(V_{10}^{\phi \beta^{T}},V_{01}^{\phi \beta^{T}},V_{00}^{\phi \beta^{T}}{)}^{T},\;V_{ab}^{\phi \beta }=\sum_{l=1}^{L}q_{l}E\;\left[\frac{\xi_{l1}}{\pi_{11,l1}}\int_{0}^{\tau }\left\{Z_{l1}-e_{l,m}\left(\beta ,t\right)dM_{l1}\left(t\right)\right\}\delta_{ab,l1}\left\{\xi_{l1}-\frac{\pi_{11,l1}}{\pi_{11,l1}+\pi_{ab,l1}}\right\}\omega_{ab,l1}^{T}\right],\;V_{m}^{\phi }=\left(\begin{array}{ccc} V_{10,10}^{\phi } & V_{10,01}^{\phi } & V_{10,00}^{\phi }\\ V_{01,10}^{\phi } & V_{01,01}^{\phi } & V_{01,00}^{\phi }\\ V_{00,10}^{\phi } & V_{00,01}^{\phi } & V_{00,00}^{\phi } \end{array}\right),\;V_{ab,a^{\prime }b^{\prime }}^{\phi }=\sum_{l=1}^{L}q_{l}E(\delta_{ab,l1}\delta_{a^{\prime }b^{\prime },l1}\left\{\xi_{l1}-\frac{\pi_{11,l1}}{\pi_{11,l1}+\pi_{ab,l1}}\right\}\left\{\xi_{l1}-\frac{\pi_{11,l1}}{\pi_{11,l1}+\pi_{a^{\prime }b^{\prime },l1}}\right\}\;\times \omega_{ab,l1}^{T}\omega_{a^{\prime }b^{\prime },l1}),\;dM_{li}\left(t\right)=dN_{li}\left(t\right)-Y_{li}\left(t\right)\exp\left(\beta^{T}Z_{li}\right)d\Lambda_{l0}\left(t\right),\;q_{l}=\underset{n\to \infty }{\lim}\;n_{l}/n.$$

*2. If* $\pi_{ab,i}$ *is known*, $\sqrt{n}\left({\hat{\beta }}_{m}-\beta_{m0}\right)$ *is asymptotically normally distributed with mean* **0** *and covariance matrix* ${\left\{I_{m}\left(\beta_{m0}\right)\right\}}^{-1}\Sigma_{m}^{\beta_{m0}}{\left\{I_{m}\left(\beta_{m0}\right)\right\}}^{-1}$.

The proof of Theorem 2 is similar to one of Theorem 1 and thus omitted. The asymptotics for more than two missing covariates can be similarly established. Now we have the main theorem on $p\left(\theta |\hat{\theta }\right)$ as follows:

**Theorem 3** *Let* $\hat{\theta }$ *be the solution to* $U_{n}\left(\theta \right)=0$. *Under Conditions C1-C17, the posterior distribution* $p\left(\theta |\hat{\theta }\right)$, *generated by the two-step method above, satisfies*

(14) $$p(\theta |\hat{\theta })\to \psi_{\hat{\theta },Var(\hat{\theta })}(\theta ),$$

(15) $$p\;\left(\lim_{n\to \infty }\int_{J_{n}\left(\theta_{0}\right)}\psi_{\hat{\theta },Var(\hat{\theta })}(\theta )d\theta \right)=1,$$

*where* $\psi_{\hat{\theta },Var\left(\hat{\theta }\right)}\left(\cdot \right)$ *is the density of normal distribution with mean* $\hat{\theta }$ *and variance* $Var\left(\hat{\theta }\right)$.

Its proof is similar to the proof of Theorem 4.1 of Sang and Kim [32] and thus is omitted. Results (14) and (15) show the convergence of the posterior distribution to a normal distribution and the posterior consistency, respectively. In particular, (14) implies the confidence region from the proposed Bayesian method is asymptotically equivalent to the frequentist confidence region based on asymptotic normality of ***θ***. Thus, our proposed Bayesian method is calibrated to frequentist inference.

The proposed Bayesian estimators for ***ϕ*** and ***β*** can be obtained by the medians of the draws from the approximated posterior distribution. Because the posterior distribution is approximately normal by Theorem 3, one can construct the confidence region using the equal-tailed credible interval (ETI) or the level-*α* Bayesian High Posterior Density credible region using $j^{*}$ defined as $C^{*}\left(\alpha \right)=\left\{\theta :P\left(\theta |\hat{\theta }\right)\geq j^{*}\left(\alpha \right)\right\}$ [7].

## 4 Simulation

We conducted two simulation studies to investigate the finite sample properties of the approximate Bayesian method and the IPW method for stratified data. We compared them with the CC method.

In the first simulation, we considered a stratified PH model with two strata, i.e., *L* = 2. Two covariates were generated for each stratum: *Z*_11_ from the Bernoulli distribution with probability 0.4 and *Z*_12_ from the standard normal distribution for stratum 1; *Z*_21_ from the Bernoulli distribution with probability 0.6 and *Z*_22_ from the normal distribution with mean 1 and standard deviation 0.7 for stratum 2. Event times were generated based on the stratified PH model (1). We considered $\lambda_{10}\left(t\right)=4t^{3}$ for stratum 1 and $\lambda_{20}\left(t\right)=2/3t^{-2/3}$ for stratum 2. We set $\beta ={\left(\beta_{1},\beta_{2}\right)}^{T}={\left\{\log\left(2\right),\log\left(2\right)\right\}}^{T}$. Independent of event times, censoring times were generated from a uniform distribution. Two overall event probabilities were examined: 50% and 70%. Some values of $Z_{l1}$ were missing and $Z_{l2}$ for *l* = 1, 2 was fully observed. The observation indicator $\xi_{li}’s$ were independently generated from the Bernoulli distribution with probability $\pi_{li}=\exp\left\{\phi_{0}+\phi_{1}I\left(l=2\right)+\phi_{2}Z_{l2}+\phi_{3}\Delta_{li}\right\}/\left\{1+\exp(\phi_{0}+\phi_{1}I\left(l=2\right)+\phi_{2}Z_{l2}+\phi_{3}\Delta_{li}\right\}$ for l=1,2, where ${\left(\phi_{0},\phi_{1},\phi_{2},\phi_{3}\right)}^{T}={\left(1.2,-1.5,0.5,-1\right)}^{T}$. The missing rates were approximately 60% for stratum 1 and 40% for stratum 2, respectively. Thus, the overall rate of missingness was 50%.

Four sample sizes were considered: *n* = 500, 1000, and 2000. Table 1 summarizes the simulation results based on *B* = 1000 Monte Carlo samples. For the proposed method, we obtained 1000 posterior medians and calculated the bias of the average of the 1000 medians, their standard deviations (*SD*), and the average percentage that 95% ETIs include the true parameters (*CR_E_*).s For IPW, CC methods, the bias of the average of mean, their average of standard errors (*SE*), and 95% coverage rates (*CR*) were calculated. As seen in Table 1, the average of the posterior medians from the approximate Bayesian method and the average of the IPW estimators were close to the true values. All standard deviations of the approximate Bayesian method are close to the average of standard errors of the IPW method. The range of the average percentages that 95% ETIs include the true parameters and the coverage rates of the IPW method are between 93% and 96%. These results are consistent with Theorem 3. Standard deviations and the average of standard errors are larger as event rate is lower or sample size is smaller. In contrast, the CC method has biases and low coverage rates ranged from 71% to 90% for completely observed covariate *Z^c^*, which are well below 95%. Furthermore, this phenomenon becomes more severe as the sample size increases.

We also conducted simulations for correlated covariates, different *β*’s, and missing rates. Table S1 of the Supplementary Material summarizes the results that are similar to Table 1. When magnitude of *β* is larger and missing rate is higher, the CC method performed worse.

In the second simulation, we considered a stratified PH model (*L* = 2) with two missing covariates. We compared the proposed methods of Section 2.4 with those in Section 2.3, and the CC method. Two covariates, *Z*_1_ and *Z*_2_ were independently generated from the Bernoulli distribution with probabilities 0.4 and 0.5 for stratum 1 and with probabilities 0.6 and 0.4 for stratum 2. One covariate *Z*_3_ was generated from a uniform distribution on [0, 1]. Event times were generated from the stratified PH model (1). We considered constant baseline hazard $\lambda_{10}\left(t\right)=1$ for stratum 1 and $\lambda_{20}\left(t\right)=2$ for stratum 2. We set $\beta ={\left(\beta_{1},\beta_{2}\right)}^{T}={\left(0.3,0.3,-0.3\right)}^{T}$ and (0.7, 0.7, −0.7)*^T^*. Independent of event times, censoring times were generated from a uniform distribution. The overall event probability was 55%: 47% for stratum 1 and 63% for stratum 2. Two covariates *Z*_1_ and *Z*_2_ were subject to missing. Define *η*_1_ and *η*_2_ be the observation indicator for *Z*_1_ and *Z*_2_, respectively. There were four possible missing categories: 1) two covariates are fully observed $\left(v_{11}=I\left(\eta_{1}=1,\eta_{2}=1\right)\right),2)$ only *Z*_2_ is missing $\left(v_{10}=I\left(\eta_{1}=1,\eta_{2}=0\right)\right),3)$ only *Z*_1_ is missing $\left(v_{01}=I\left(\eta_{1}=0,\eta_{2}=1\right)\right)$, 4) both *Z*_1_ and *Z*_2_ are missing $\left(v_{00}=I\left(\eta_{1}=0,\eta_{2}=0\right)\right)$. Missing indicator *v*_*ab*_’s were independently generated from the multinomial distribution with probability $\pi_{ab,i}=\exp\left\{\phi_{ab}^{T}\omega_{ab}\right\}/\left(1+\sum_{a=0}^{1}\sum_{b=0}^{1}\exp\left\{\phi_{ab}^{T}\omega_{ab}\right\}\right)$ for a,b=0,1, where $\phi_{10}={\left(\phi_{10,0},\phi_{10,1},\phi_{10,2},\phi_{10,3},\phi_{10,4}\right)}^{T}={\left(-3,2,3,-2,-2\right)}^{T}$, $\phi_{01}={\left(\phi_{01,0},\phi_{01,1},\phi_{01,2},\phi_{01,3},\phi_{01,4}\right)}^{T}={\left(-1.2,-2,2,-0.2,0.1\right)}^{T}$, $\phi_{00}={\left(\phi_{00,0},\phi_{00,1},\phi_{00,2},\phi_{00,3}\right)}^{T}={\left(0.3,-1.5,-0.9,0.1\right)}^{T}$, $\omega_{10}={\left(1,\Delta ,Z_{1},Z_{3},I\left(l=2\right)\right)}^{T}$, $\omega_{01}={\left(1,\Delta ,Z_{2},Z_{3},I\left(l=2\right)\right)}^{T}$, and $\omega_{00}={\left(1,\Delta ,Z_{3},I\left(l=2\right)\right)}^{T}$. Then, overall missing probabilities for $v_{11}$, $v_{10}$, $v_{01}$, and $v_{00}$ were 17%, 13%, 20%, and 50%, respectively: 17%, 14%, 29%, and 40% for stratum 1 and 17%, 11%, 12%, and 60% for stratum 2. Two sample sizes were examined: *n* = 1000 and 1500.

Table 2 shows that estimates for the proposed approximate Bayesian and IPW methods for multiple missing patterns are approximately unbiased. The average percentages of 95% ETIs and coverage rates of the IPW method are close to nominal level 95%. However, the CC method and the approximate Bayesian method/IPW for a single missing pattern have biases, where the coverage rates for the missing covariates $Z_{1}^{m}$ and $Z_{2}^{m}$ are from 60% to 87%. When the sample size increases, coverage rates become lower and further away from 95%.

## 5 Real data application

We applied the proposed approximate Bayesian and IPW methods to the following two registry data sets: 1) the stem cell transplantation (HCT) data which Dreger et al. [11] analyzed to study patients with DLBCL; 2) the HCT data which Pidala et al. [26] investigated to study patients with myelodysplastic syndrome (MDS). The DLBCL data and the MDS data are for the stratified PH model with a single missing covariate and with two missing covariates, respectively. Thus, we applied the methods of Section 2.2 and 2.3 to the DLBCL data, and we used the methods of Section 2.4 for the MDS data.

### 5.1 Stratified PH model with a single missing covariate

The DLBCL data [11] consisted of 1,394 adult patients. Overall survival was an outcome of interest for the analysis. The number of patients who died and were censored are 725 (52%) and 669 (48%), respectively. Among 1394 patients, there are 127 patients (9%) with (haplo-HCT); 509 patients (37%) with MSD; 488 patients (28%) with MUD with T-cell depletion; and 370 patients (26%) with MUD without T-cell depletion. In HCT studies, clinicians are often interested in evaluating the effects of remission status at time of HCT (Complete, partial, refractory), age groups (18–49, 50–59, >60), year of transplant (2008–2010, 2011–2012, 2013–2015), hematopoietic cell transplant-comorbidity index (HCT-CI) (0, 1–2, ≥3), and Karnofsky performance score (<90, ≥90) on the outcome due to their clinical importance (Kumar et al. [21], Papanicolaou et al. [25], and Ustun et al. [37]). Thus, we adjusted these five covariates in the model. Four hundred eighty four patients (35%) have missing values in HCT-CI. We tested the PH assumption for each covariate by testing whether the coefficient of log *t* × *Z* is equal to zero for each variable [20]. Remission status at time of transplant did not satisfy the PH assumption at a significant level 0.05 (*p*-value = 0.0047). Thus, we stratified the PH model according to remission status.

We fitted the logistic regression to obtain the propensity score by allowing a different intercept for each stratum. Six variables including the stratum variable, year of transplant, age group, donor type, death indicator, and time to death were statistically significant at a significance level 0.05. We used these six variables to calculate propensity scores. We fitted the approximate Bayesian method, IPW method, and the CC method.

Table 3 reports the analysis result including i) the posterior median ${\tilde{\beta }}^{m}$ and 95% ETI (ETI) for the approximate Bayesian method; ii) $\hat{\beta }$ and its 95% confidence intervals for the IPW method and the CC method. As expected, the results from the approximate Bayesian method and the IPW method were similar. However, the 95% confidence intervals of the IPW method are slightly wider than the 95% ETIs of the approximate Bayesian method in general. On the other hand, the effects of donor group, HCT-CI score, and Karnofsky score from the approximate Bayesian method and the IPW method were similar to those from the CC method. However, the effects of year of transplant were different: based on the approximate Bayesian method and the IPW method, patients who had HCT from 2008 to 2010 were more likely to die after HCT than those who had HCT from 2013 to 2015. The results from the approximate Bayesian/IPW methods and CC methods are different in donor group and year of transplant. In particular, although year of transplant did not reach statistical significance in the model from CC methods, the parameter estimates for 2008-2012 were negative. Thus, the results from CC methods imply patients who got transplant in recent years (2013-2015) experienced worse survival than earlier years (2008-2012). It is common in DLBCL studies that the progress of patients who got HCT in recent years was better than those who got HCT in earlier years [22,2,33]. Thus, the results on year of transplant from the CC method are counter-intuitive. In contrast with these, the year of transplant effects from the approximate Bayesian method and the IPW method are consistent with the current medical literature. The effects of year of transplant and HCT-CI contradict the current HCT literature.

We conducted a sensitive analysis by examining various propensity score models to investigate the missing-not-at-random assumption. We observed that the progress of patients who got HCT in recent years was worse than those who got HCT in earlier years which is inconsistent with the current HCT literature. Thus, the MAR assumption appears to be reasonable for this data set.

### 5.2 Stratified PH model with two missing covariates

The MDS data [26] for the analysis consisted of 787 adults or children with diagnoses of MDS, who underwent first myeloablative-unrelated bone marrow or peripheral blood stem cell transplantation conducted between 1999 and 2011 patients. An outcomes of interest in this analysis was an overall survival. The number of events and censoring are 418 (53%) and 369 (47%), respectively. Two covariates including HLA-DPB1 classification according to T-cell epitope grouping (HLAD) and KPS have missing values. The original analysis which Pidala et al. [26] conducted excluded patients with HLAD missing from the analysis. The number of patients who had missing values in both HLAD and KPS, only HLAD, only KPS is 17, 330, and 45, respectively. The overall missing rate is about 50% (= 392/787 × 100). Among 787 patients, there are 64 patients (8%) with fully matched HLAD; 229 patients (29%) with Permissive HLAD; 67 patients (9%) with GvH non-permissive HLAD; 80 patients (10%) with HvG non-permissive HLAD; and 347 patients (44%) with missing HLAD. The covariates of interest include graft type (Bone marrow, Peripheral blood), race (Caucasian, others), age groups (< 20, 20—49,>50), year of transplant (1999–2002, 2003–2006, 2007–2011), and KPS (<90, ≥90). We tested the PH assumption for each covariate by testing whether the coefficient of log *t* × *Z* is equal to zero for each variable [20]. Graft type and year of transplant were not satisfied the PH assumption at a significant level 0.05. Thus, we fitted stratified PH model. We used the approximate Bayesian method/IPW method for multiple missing patterns of Section 2.4 and compared with the approximate Bayesian method/IPW method for a single missing pattern of Section 2.2 and 2.3, and the CC method. While none of covariates were significant for two missing categories for 1) only KPS missing; 2) both HLAD and KPS in the propensity score model at the significance level 0.05, three variables including year of transplant, death indicator, and time to death were significant in the propensity score model for only HLAD missing. Thus, applying the method for multiple missing patterns appeared to be more appropriate than using that for a single missing pattern.

Table 4 reports the analysis results including i) the posterior median ${\tilde{\beta }}^{m}$ and its 95% ETI (ETI) for the approximate Bayesian method with multiple missing patterns and a single missing pattern; ii) $\hat{\beta }$ and its 95% confidence intervals for the IPW method, and the CC method. Results of approximate Bayesian/IPW methods with multiple missing patterns and a single missing pattern for KPS and race were similar. However, HLAD and age group show difference in results between the approaches for multiple missing patterns and those for a single missing pattern. While the effect of permissive classification group compared with fully matched classification group is positive from the approximate Bayesian/IPW methods for multiple missing patterns, it is negative from the approximate Bayesian/IPW methods for single missing. The results from the approximate Bayesian/IPW methods for multiple missing pattern show that the 95% CI and ETI of age 20-49 group did not contain 0, but the 95% CI and ETI of age 20-49 group contained 0 when considering a single missing pattern. The results for the CC method show the 95% CI and ETI of race other group did not contain 0 while those from the approximate Bayesian/IPW methods for multiple missing patterns contained 0.

## 6 Concluding Remarks

We have proposed new approximate Bayesian and IPW methods for the stratified PH model with incomplete covariate information. In particular, we studied multiple missing patterns, which is largely ignored in the current literature. Using the flat prior, the proposed Bayesian method is asymptotically equivalent to the frequentist IPW inference using Taylor linearization. The proposed Bayesian method can improve its performance if the prior is informative. In this case, it may be more efficient than the frequentist IPW method. The approximate Bayesian method can be further improved by adding an augmented term to the score function for the stratified PH model similarly to Wang and Chen [39] and Xu et al. [41].

The scheme of the proposed methods can also be applied to competing risks data. Exploring the cause-specific hazards model [28] and the proportional subdistribution hazards model [12] will be an interesting research topic. We only studied missing covariates in this article. In HCT studies, it is common that a portion of either time to event or outcome indicators are also missing. Handling missingness in such outcomes would be an important research problem. For causal inference, the IPW approach is widely used to adjust for the probability of treatment assignments in observational studies. Applying the proposed Bayesian method to causal inference would be a worthy future topic. The proposed methods require specifying the propensity score model correctly. One can consider nonparametric regression for the propensity score estimation or a doubly robust estimator [30] for robust estimation. Pursuing this direction would be an important research topic in the future.

## Supplementary Material

1782160_Sup_info

## Figures and Tables

**Table 1:** Simulation results for stratified survival data

		Event	Proposed method			IPW Method			CC method		
	*n*	rate	bias	*SD*	*CR_E_*	bias	*SE*	*CR*	bias	*SE*	*CR*
*Z^m^*	500	50%	0.010	0.238	0.95	0.007	0.231	0.95	0.076	0.247	0.95
		70%	0.003	0.200	0.95	0.003	0.197	0.94	0.055	0.200	0.95
	1000	50%	0.009	0.165	0.95	0.008	0.162	0.95	0.077	0.172	0.94
		70%	0.000	0.139	0.94	0.000	0.138	0.94	0.052	0.139	0.93
	2000	50%	0.003	0.114	0.94	0.002	0.113	0.95	0.070	0.120	0.92
		70%	0.000	0.097	0.95	0.000	0.096	0.96	0.050	0.097	0.92
*Z^c^*	500	50%	0.027	0.175	0.93	0.025	0.169	0.93	0.115	0.177	0.90
		70%	0.015	0.133	0.93	0.016	0.130	0.93	0.097	0.130	0.88
	1000	50%	0.006	0.121	0.94	0.005	0.118	0.94	0.094	0.122	0.88
		70%	0.001	0.092	0.95	0.001	0.091	0.95	0.085	0.090	0.85
	2000	50%	0.005	0.085	0.94	0.004	0.083	0.94	0.090	0.085	0.82
		70%	0.003	0.065	0.95	0.003	0.064	0.95	0.085	0.063	0.71

*SD*, standard deviations; *SE*, average of standard errors; *CR_E_*: the equal-tailed credible interval confidence region; *CR*, 95% coverage rates; IPW, inverse-probability-weighted; CC, complete-case.

**Table 2:** Simulation results for multiple missing patterns

			MM Proposed method			MM IPW			CC method		
n	*β*	Covariates	bias	*SD*	*CR_E_*	bias	*SE*	*CR*	bias	*SE*	*CR*
1000	0.3	$Z_{1}^{m}$	0.001	0.118	0.95	0.001	0.118	0.95	−0.122	0.103	0.78
	0.3	$Z_{2}^{m}$	0.005	0.117	0.95	0.005	0.116	0.95	0.048	0.099	0.93
	−0.3	*Z^c^*	−0.006	0.207	0.93	−0.006	0.199	0.93	0.041	0.171	0.94
	0.7	$Z_{1}^{m}$	0.007	0.121	0.94	0.007	0.119	0.94	−0.144	0.106	0.72
	0.7	$Z_{2}^{m}$	0.012	0.119	0.94	0.012	0.118	0.94	0.015	0.102	0.96
	−0.7	*Z^c^*	−0.012	0.207	0.94	−0.012	0.200	0.94	0.067	0.174	0.92
1500	0.3	$Z_{1}^{m}$	0.003	0.095	0.94	0.003	0.097	0.95	−0.123	0.084	0.69
	0.3	$Z_{2}^{m}$	0.002	0.097	0.94	0.002	0.096	0.94	0.047	0.081	0.91
	−0.3	*Z^c^*	−0.005	0.166	0.95	−0.005	0.164	0.96	0.041	0.138	0.94
	0.7	$Z_{1}^{m}$	0.004	0.100	0.93	0.004	0.098	0.94	−0.148	0.086	0.60
	0.7	$Z_{2}^{m}$	0.002	0.099	0.94	0.002	0.097	0.94	0.012	0.083	0.94
	−0.7	*Z^c^*	−0.012	0.172	0.94	−0.011	0.165	0.94	0.067	0.141	0.92

			SM Proposed method			SM IPW		
n	*β*	Covariates	bias	*SD*	*CR_E_*	bias	*SE*	*CR*
1000	0.3	$Z_{1}^{m}$	−0.150	0.115	0.72	−0.150	0.113	0.75
	0.3	$Z_{2}^{m}$	0.102	0.111	0.83	0.101	0.107	0.84
	−0.3	*Z^c^*	0.069	0.197	0.99	0.069	0.186	0.92
	0.7	$Z_{1}^{m}$	−0.143	0.115	0.74	−0.143	0.114	0.75
	0.7	$Z_{2}^{m}$	0.085	0.109	0.88	0.085	0.107	0.87
	−0.7	*Z^c^*	0.063	0.194	0.99	0.063	0.186	0.92
1500	0.3	$Z_{1}^{m}$	−0.151	0.093	0.61	−0.151	0.093	0.63
	0.3	$Z_{2}^{m}$	0.102	0.090	0.78	0.102	0.088	0.79
	−0.3	*Z^c^*	0.072	0.155	0.99	0.072	0.152	0.91
	0.7	$Z_{1}^{m}$	−0.146	0.096	0.64	−0.147	0.093	0.64
	0.7	$Z_{2}^{m}$	0.081	0.090	0.85	0.080	0.088	0.84
	−0.7	*Z^c^*	0.067	0.159	0.99	0.067	0.152	0.92

MM, multiple missing patterns; SM, a single missing pattern; IPW, inverse-probability-weighted; CC, complete-case; *SD*, standard deviations; *SE*, average of standard errors; *CR_E_*: the equal-tailed credible interval confidence region; *CR*, 95% coverage rates.

**Table 3:** Stem cell transplantation data analysis for stratified survival data with a single missing covariate

	Approximate Bayesian		IPW method		CC method	
Variable	${\tilde{\beta }}^{m}$	95 % ETI	$\hat{\beta }$	95 % CI	$\hat{\beta }$	95 % CI
**Donor group**						
HD (ref)	0		0		0	
MSD	−0.141	( −0.434 , 0.193 )	−0.143	( −0.450 , 0.164 )	−0.134	( −0.400 , 0.132 )
MUD WTD	0.052	( −0.262 , 0.401 )	0.053	( −0.282 , 0.387 )	0.038	( −0.236 , 0.313 )
MUD WOTD	−0.092	( −0.385 , 0.252 )	−0.088	( −0.411 , 0.235 )	−0.132	( −0.407 , 0.144 )
**Year of transplant**						
2013-2015 (ref)	0		0		0	
2008-2010	0.231	( 0.012 , 0.443 )	0.222	( 0.009 , 0.435 )	−0.036	( −0.222 , 0.150 )
2011-2012	0.158	( −0.070 , 0.378 )	0.152	( −0.076 , 0.381 )	−0.043	( −0.232 , 0.146 )
**Age group**						
18-49 (ref)	0		0		0	
50-59	0.104	( −0.127 , 0.336 )	0.105	( −0.130 , 0.339 )	0.130	( −0.055 , 0.316 )
>60	0.202	( −0.027 , 0.436 )	0.202	( −0.028 , 0.433 )	0.205	( 0.014 , 0.396 )
**HCT-CI**						
0 (ref)	0		0		0	
1-2	0.029	( −0.204 , 0.270 )	0.030	( −0.207 , 0.267 )	0.041	( −0.178 , 0.259 )
≥ 3	0.215	( 0.001 , 0.441 )	0.217	( −0.004 , 0.438 )	0.244	( 0.032 , 0.455 )
**Karnofsky score**						
≥ 90 (ref)	0		0		0	
< 90	0.198	( 0.010 , 0.387 )	0.199	( 0.013 , 0.385 )	0.274	( 0.120 , 0.429 )

IPW, inverse-probability-weighted; CC, complete-case; ref, reference group; ETI, equal-tailed credible interval; HD, Haploidentical donors; MSD, matched sibling donors; MUD WTD, matched unrelated donors with T-cell depletion; MUD WOTD, matched unrelated donors without T-cell depletion.

**Table 4:** Stem cell transplantation data analysis for multiple missing patterns

	MM Approximate Bayesian		MM IPW method		CC method	
Variable	${\tilde{\beta }}^{m}$	95 % ETI	$\hat{\beta }$	95% CI	$\hat{\beta }$	95% CI
**HLAD**						
Fully matched (ref)	0		0		0	
Permissive	0.076	( −0.297 , 0.536 )	0.081	( −0.317 , 0.480 )	−0.049	( −0.445 , 0.347 )
GvH non-permissive	0.354	( −0.182 , 0.845 )	0.344	( −0.151 , 0.839 )	0.286	( −0.182 , 0.753 )
HvG non-permissive	0.080	( −0.465 , 0.595 )	0.081	( −0.419 , 0.581 )	0.063	( −0.396 , 0.522 )
**Karnofsky score**						
90 - 100% (ref)	0		0		0	
< 90%	0.450	( 0.123 , 0.738 )	0.446	( 0.136 , 0.755 )	0.443	( 0.157 , 0.728 )
**Race**						
Caucasian	0		0		0	
others	0.440	( −0.235 , 0.898 )	0.438	( −0.088 , 0.963 )	0.483	( 0.018 , 0.948 )
**Age group**						
< 20 (ref)	0		0		0	
20-49	0.520	( 0.085 , 1.079 )	0.522	( 0.054 , 0.990 )	0.582	( 0.122 , 1.042 )
> 50	1.039	( 0.627 , 1.606 )	1.034	( 0.546 , 1.522 )	1.058	( 0.586 , 1.530 )

	SM Approximate Bayesian		SM IPW method	
Variable	${\tilde{\beta }}^{m}$	95 % ETI	$\hat{\beta }$	95% CI
**HLAD**				
Fully matched (ref)	0		0	
Permissive	−0.014	( −0.506 , 0.582 )	−0.036	( −0.580 , 0.509 )
GvH non-permissive	0.270	( −0.369 , 0.997 )	0.309	( −0.348 , 0.965 )
HvG non-permissive	0.061	( −0.791 , 0.723 )	0.062	( −0.608 , 0.732 )
**Karnofsky score**				
90 - 100% (ref)	0		0	
< 90%	0.454	( 0.107 , 0.782 )	0.446	( 0.023 , 0.870 )
**Race**				
Caucasian	0		0	
others	0.537	( −0.553 , 1.062 )	0.466	( −0.272 , 1.205 )
**Age group**				
< 20 (ref)	0		0	
20-49	0.558	( −0.072 , 1.174 )	0.560	( −0.024 , 1.145 )
> 50	1.099	( 0.491 , 1.692 )	1.035	( 0.408 , 1.661 )

MM, multiple missing pattern; SM, single missing pattern; IPW, inverse-probability-weighted; CC, complete-case; ETI, equal-tailed credible interval; TX, transplant; HLAD, DPB1 classification according to T-cell epitope grouping; ref, reference group.

## Appendix

We derive ***Σ*** and its estimator in the Appendix. Let $dM_{li}\left(t\right)=dN_{li}\left(t\right)-Y_{li}\left(t\right)\exp\left\{\beta^{T}Z_{li}\right\}d\Lambda_{l}\left(t\right)$. The posterior distribution is

(16)
$$p\left(\eta |U_{n}\right)∼N\;\left[\left(\begin{array}{l} 0\\ 0 \end{array}\right),\frac{\Sigma }{n}=\left(\begin{array}{cc} Var\left(U_{1}\right) & Cov\left(U_{1},U_{2}\right)\\ Cov\left(U_{1},U_{2}\right) & Var\left(U_{2}\right) \end{array}\right)\right],$$

where

$$\;Var\left\{U_{1}(\beta ,\phi )\right\}\;=Var\left[n^{-1}\overset{L}{\underset{l=1}{\sum }}\overset{n_{l}}{\underset{i=1}{\sum }}\frac{\xi_{li}}{\pi_{li}}\int_{0}^{\tau }\left\{Z_{li}-\frac{S_{l}^{\left(1\right)}(\beta ,t)}{S_{l}^{\left(0\right)}(\beta ,t)}\right\}dM_{li}(t)\right]\;=Var\left[n^{-1}\overset{L}{\underset{l=1}{\sum }}\overset{n_{l}}{\underset{i=1}{\sum }}\frac{\xi_{li}}{\pi_{li}}\int_{0}^{\tau }\left\{Z_{li}-e_{l}(\beta ,t)\right\}dM_{li}(t)\right]\;=E\;{\left[n^{-2}\overset{L}{\underset{l=1}{\sum }}\overset{n_{l}}{\underset{i=1}{\sum }}\frac{\xi_{li}}{\pi_{li}}\int_{0}^{\tau }\left\{Z_{li}-e_{l}(\beta ,t)\right\}dM_{li}(t)\right]}^{⊗2}.$$

We can estimate $Var\left\{U_{1}\left(\beta ,\phi \right)\right\}$ given β and ϕ as follows:

$$\hat{Var}\left\{U_{1}(\beta ,\phi )\right\}=\frac{1}{n^{2}}\overset{L}{\underset{l=1}{\sum }}\overset{n_{l}}{\underset{i=1}{\sum }}\frac{\xi_{li}}{\pi_{li}^{2}}\int_{0}^{\tau }{\left[\left\{Z_{li}-\frac{S_{l}^{\left(1\right)}(\beta ,t)}{S_{l}^{\left(0\right)}(\beta ,t)}\right\}\;\left\{dN_{li}(t)-d{\hat{\Lambda }}_{0l}(t)\right\}\right]}^{⊗2},$$

where $d{\hat{\Lambda }}_{0l}\left(t\right)=\sum_{i=1}^{n_{l}}dN_{li}\left(t\right)/n_{l}S_{l}^{\left(0\right)}\left(\beta ,t\right)$.

We can obtain $\hat{Var}\left(U_{2}\right)$ given β and ϕ as follows:

$$\hat{Var}\left(U_{2}\right)=\frac{1}{n^{2}}\overset{L}{\underset{l=1}{\sum }}\overset{n_{l}}{\underset{i=1}{\sum }}\pi_{li}\left(1-\pi_{li}\right)\omega_{li}\omega_{li}^{T}.$$

Next, $Cov\left(U_{1},U_{2}\right)$ given β and ϕ can be estimated by

$$\hat{Cov}\left(U_{1},U_{2}\right)=\hat{Cov}[\frac{1}{n}\overset{L}{\underset{l=1}{\sum }}\overset{n_{l}}{\underset{i=1}{\sum }}\frac{\xi_{li}}{\pi_{li}}\int_{0}^{\tau }\left\{Z_{li}-\frac{S_{l}^{\left(1\right)}(\beta ,t)}{S_{l}^{\left(0\right)}(\beta ,t)}\right\}\;dM_{li}(t),\;\;\;\;\;\;\;\;\frac{1}{n}\overset{L}{\underset{l=1}{\sum }}\overset{n_{l}}{\underset{i=1}{\sum }}\left\{\xi_{li}-\pi_{li}\right\}\omega_{li}^{T}]\;\;\;\;\;\;\;\;=\frac{1}{n^{2}}\overset{L}{\underset{l=1}{\sum }}\overset{n_{l}}{\underset{i=1}{\sum }}\frac{\xi_{li}\left(1-\pi_{li}\right)}{\pi_{li}}\int_{0}^{\tau }\left\{Z_{li}-\frac{S_{l}^{\left(1\right)}(\beta ,t)}{S_{l}^{\left(0\right)}(\beta ,t)}\right\}\;dM_{li}(t)\times \omega_{li}^{T}.$$

The estimator $\hat{\Sigma }$ is

(17)
$$\frac{\hat{\Sigma }}{n}=\left(\begin{array}{cc} \hat{Var}\left(U_{1}\right) & \hat{Cov}\left(U_{1},U_{2}\right)\\ \hat{Cov}\left(U_{1},U_{2}\right) & \hat{Var}\left(U_{2}\right) \end{array}\right).$$
