# Supplementary material for: A calibrated Bayesian method for the stratified proportional hazards model with missing covariates
Source: Lifetime Data Anal. Author manuscript; Available in PMC 2023 Apr 1. (PMC8977246; doi:10.1007/s10985-021-09542-4)
Supplement: 1782160_Sup_info [file NIHMS1782160-supplement-1782160_Sup_info.pdf]

Supplementary Materials

1 Appendix A: Additional simulation results

We conducted an additional simulation study for correlated covariates, different βs, and missing rates. In this simulation, we considered a stratified PH model with two strata, i.e., $L = 2$. Two covariates were generated for each stratum: Z_{11} from the normal distribution with mean 0 and standard deviation 1 and Z_{12} from the normal distribution with mean 0.5 and standard deviation 1 for stratum 1; Z_{21} from the normal distribution with mean 0 and standard deviation 1 and Z_{22} from the normal distribution with mean 0.7 and standard deviation 1 for stratum 2. We considered two correlation sets: i) 0.1 for stratum 1 and 0.3 stratum 2, ii) 0.5 for stratum 1 and 0.7 stratum 2. Event times were generated based on the stratified PH model. We considered constant baseline hazard function such that $\lambda_{10}(t) = 1$ for stratum 1 and $\lambda_{20}(t) = 2$ for stratum 2. We set $\boldsymbol{\beta} = (\beta_1, \beta_2)^T = \{0.5, -0.5\}^T$ and $(\beta_1, \beta_2)^T = \{1.5, -1.5\}^T$. Independent of event times, censoring times were generated from a uniform distribution. We considered overall event probabilities with 50%. Some values of Z_{l1} were missing and Z_{l2} for $l = 1, 2$ was fully observed. The observed indicator ξ_{li}'s were independently generated from the Bernoulli distribution with probability $\pi_{li} = \exp\{\phi_0 + \phi_1 I(l = 2) + \phi_2 Z_{l2} + \phi_3 \Delta_{li}\}/\{1 + \exp(\phi_0 + \phi_1 I(l = 2) + \phi_2 Z_{l2} + \phi_3 \Delta_{li}\}$ for $l = 1, 2$, where $(\phi_0, \phi_1, \phi_2, \phi_3)^T = (1.2, -1.5, 0.5, -1)^T$ and $(\phi_0, \phi_1, \phi_2, \phi_3)^T = (2.5, -1.5, 0.5, -1)^T$. When $(\phi_0, \phi_1, \phi_2, \phi_3)^T = (1.2, -1.5, 0.5, -1)^T$, the missing rates were approximately 40% for stratum 1 and 60% for stratum 2, respectively. Thus, the overall missing rates was 50%. When $(\phi_0, \phi_1, \phi_2, \phi_3)^T = (2.5, -1.5, 0.5, -1)^T$, the missing rates were approximately 15% for stratum

1 and 35% for stratum 2, respectively. Thus, the overall missing rates was 25%. The sample size was 1000.

Table S1 summarizes the results based on $B = 1000$ Monte Carlo samples. For the proposed method, we obtained 1000 posterior medians and calculated the bias of the average of the 1000 medians, their standard deviations (SD), and the average percentage that 95% ETIs include the true parameters (CR_E). The results show that they were similar to Table 1 in the manuscript. When magnitude of β is larger and missing rate is higher, and CC performed worse.

Table S1: Simulation results for stratified survival data with different correlations, missing rates, and β

	corr	β	Missing rate	Proposed method bias	Proposed method SD	Proposed method CR_E	IPW Method bias	IPW Method SE	IPW Method CR	CC method bias	CC method SE	CC method CR
Z^m	(0.1,0.3)	(0.5,-0.5)	50%	0.009	0.082	0.93	0.009	0.081	0.93	0.071	0.085	0.86
			25%	0.003	0.060	0.93	0.003	0.059	0.93	0.036	0.062	0.91
		(1.5,-1.5)	50%	0.015	0.124	0.93	0.015	0.122	0.94	0.142	0.123	0.81
			25%	0.007	0.088	0.94	0.007	0.087	0.94	0.068	0.089	0.88
	(0.5,0.7)	(0.5,-0.5)	50%	0.009	0.097	0.93	0.010	0.096	0.94	0.072	0.101	0.89
			25%	0.003	0.072	0.94	0.004	0.071	0.95	0.037	0.075	0.92
		(1.5,-1.5)	50%	0.011	0.131	0.94	0.013	0.129	0.95	0.157	0.131	0.78
			25%	0.008	0.095	0.94	0.008	0.094	0.93	0.081	0.097	0.88
Z^c	(0.1,0.3)	(0.5,-0.5)	50%	-0.008	0.084	0.94	-0.009	0.084	0.95	-0.005	0.088	0.95
			25%	-0.002	0.061	0.94	-0.002	0.061	0.95	0.006	0.064	0.95
		(1.5,-1.5)	50%	-0.017	0.124	0.94	-0.017	0.122	0.94	-0.091	0.126	0.90
			25%	-0.007	0.088	0.93	-0.007	0.088	0.93	-0.036	0.091	0.93
	(0.5,0.7)	(0.5,-0.5)	50%	-0.009	0.099	0.95	-0.009	0.098	0.96	-0.006	0.103	0.95
0.029	0.073	0.92	-0.099	0.070	0.70							
			25%	-0.002	0.073	0.95	-0.002	0.073	0.96	0.006	0.077	0.97
		(1.5,-1.5)	50%	-0.016	0.131	0.94	-0.017	0.129	0.95	-0.103	0.134	0.89
			25%	-0.007	0.095	0.94	-0.007	0.095	0.95	-0.044	0.099	0.92

CR_E: ETI confidence region
